# Surgical Models of Liver Regeneration in Pigs: A Practical Review of the Literature for Researchers

**DOI:** 10.3390/cells12040603

**Published:** 2023-02-13

**Authors:** Lorenzo Cinelli, Edoardo Maria Muttillo, Emanuele Felli, Andrea Baiocchini, Fabio Giannone, Jacques Marescaux, Didier Mutter, Michel De Mathelin, Sylvain Gioux, Eric Felli, Michele Diana

**Affiliations:** 1 Department of Gastrointestinal Surgery, San Raffaele Hospital IRCCS, 20132 Milan, Italy; 2 Research Institute against Digestive Cancer (IRCAD), 67000 Strasbourg, France; 3 Division of General Surgery, Department of Medical and Surgical Sciences and Translational Medicine, Sant’Andrea University Hospital, Sapienza University of Rome, Via di Grottarossa 1035, 00189 Rome, Italy; 4 Service Chirurgie Digestive et Transplantation Hépatique, Hôpital Trousseau CHU, 37170 Tours, France; 5 Department of Pathology, San Camillo Forlanini Hospital, 00152 Rome, Italy; 6 Digestive and Endocrine Surgery, Nouvel Hopital Civil, University of Strasbourg, 67000 Strasbourg, France; 7 Institut de Chirurgie Guidée par L’image, University Hospital Institute (IHU), University of Strasbourg, 67000 Strasbourg, France; 8 ICube Laboratory, Photonics Instrumentation for Health, 67400 Strasbourg, France; 9 Department of Visceral Surgery and Medicine, Inselspital, Bern University Hospital, University of Bern, 3012 Bern, Switzerland

**Keywords:** liver injury, liver regeneration, liver repair, hepatotoxicity, liver diseases

## Abstract

The remarkable capacity of regeneration of the liver is well known, although the involved mechanisms are far from being understood. Furthermore, limits concerning the residual functional mass of the liver remain critical in both fields of hepatic resection and transplantation. The aim of the present study was to review the surgical experiments regarding liver regeneration in pigs to promote experimental methodological standardization. The Pubmed, Medline, Scopus, and Cochrane Library databases were searched. Studies evaluating liver regeneration through surgical experiments performed on pigs were included. A total of 139 titles were screened, and 41 articles were included in the study, with 689 pigs in total. A total of 29 studies (71% of all) had a survival design, with an average study duration of 13 days. Overall, 36 studies (88%) considered partial hepatectomy, of which four were an associating liver partition and portal vein ligation for staged hepatectomy (ALPPS). Remnant liver volume ranged from 10% to 60%. Only 2 studies considered a hepatotoxic pre-treatment, while 25 studies evaluated additional liver procedures, such as stem cell application, ischemia/reperfusion injury, portal vein modulation, liver scaffold application, bio-artificial, and pharmacological liver treatment. Only nine authors analysed how cytokines and growth factors changed in response to liver resection. The most used imaging system to evaluate liver volume was CT-scan volumetry, even if performed only by nine authors. The pig represents one of the best animal models for the study of liver regeneration. However, it remains a mostly unexplored field due to the lack of experiments reproducing the chronic pathological aspects of the liver and the heterogeneity of existing studies.

## 1. Introduction

During the last decades, indications for liver resections increased due to more aggressive and multimodal treatment of primary and secondary liver malignancies [1,2]. Furthermore, improvements in surgical techniques, anaesthesiology, and postoperative care, have made human liver transplantation more feasible and safer since the first successful liver transplantation in 1967 [3]. Several new procedures have been developed, such as reduced, split, and living-related liver transplantation [4]. Both resection and transplantation have to face dangerous limits concerning the functional mass of the liver. Remnant liver volume (RLV) < 25% after major hepatectomies [5] or too small a volume of the transplanted graft (graft weight/body weight ratio < 0.8%) [6] lead to life-threatening conditions known as post-hepatectomy liver failure [7] and “small-for-size” syndrome [8], respectively. The need to improve the limit of RLV has stimulated great interest in new methods aimed at increasing the rate of the hepatic regenerative process during the last decade. New techniques such as portal vein embolization (PVE), portal vein ligation (PVL), and associating liver partition and portal vein ligation for staged hepatectomy (ALPPS) were developed over the years and gained crucial clinical relevance. This topic has a long path, starting with Higgins and Anderson, who performed, in 1931, a standardized partial hepatectomy (PH) on rats removing two-thirds of the total liver. They described for the first time a model of compensatory surgically induced hyperplasia [9]. Grindlay et al. [10] studied regeneration after PH on dogs in 1952, while the first porcine model appeared in 1976, thanks to Gallot et al. [11]. Rous and Larimore demonstrated the effects of selective PVL on dogs for the first time in 1920 [12], and the PVE was applied to humans only in the 1980s [13]. Finally, ALPPS was first described in 2012 by Schnitzbauer et al. [14]. The complete knowledge of the mechanisms involved in liver regeneration and their consequent replication could be revolutionary in the field of surgery. The main benefits could result in a shorter waiting list using small partial grafts of the same liver (from cadaveric or living donors) [6] and the ability of the surgeons to perform even more major hepatectomies, widening the treatment options to beat liver pathologies. Although the liver is an organ with an extraordinary capacity to regenerate upon various injuries, as known since the ancient Greek myth of Prometheus [9], its regenerative potential, as well as its mechanisms, are still not well understood. Liver regeneration should be considered a complex multimodal functional compensatory hyperplasia, but it does not recapitulate liver organogenesis. Its process can be divided into three important distinctive phases including: (i) initial hypertrophy preparing the liver cells for replication providing an overexpression of specific genes; (ii) hyperplasia with a series of cycles of cell division and expansion; (iii) termination phase which stops the regenerative process and prevents liver overgrowth [15]. These mechanisms are activated and regulated by important mediators, such as cytokines, directly expressed at the site of injury and also migrated into the liver via the circulatory system [16]. A deep knowledge of liver regeneration would help in the prediction of the outcome, which ultimately could be useful for the precision medicine approach, adapting the surgical technique to each specific clinical case. For this reason, animal experimentation is still crucial.

Most knowledge regarding the pathophysiological basis of liver regeneration has been derived from rodents. However, the small body size of mice has limited their application in investigating human diseases, and it is difficult to obtain large numbers of humanized hepatocytes from mouse models [17]. Hence, the growing interest in larger experimental models to study liver regeneration. Pigs and humans have anatomical, cellular and physiological similarities that make the porcine experimental model the most suitable one. Phylogenetically, pigs are threefold closer to humans on the nucleotide level than are mice [18]. The macroscopic structure (same subdivision in lobes and segments of the human liver) and the vascularization are comparable to humans. The absence of communications between the right and left portal branches allows for detailed studies on major liver resections [19]. Moreover, the immune system of pigs is similar to that of humans, and some inbred pigs are useful for reproducible studies of physiologic and immunologic mechanisms thanks to their genetically defined and fixed major histocompatibility complex [20]. The present review aims at providing a useful guide for researchers who want to study liver regeneration by using surgical experiments on pigs.

## 2. Materials and Methods

**Figure 1:**
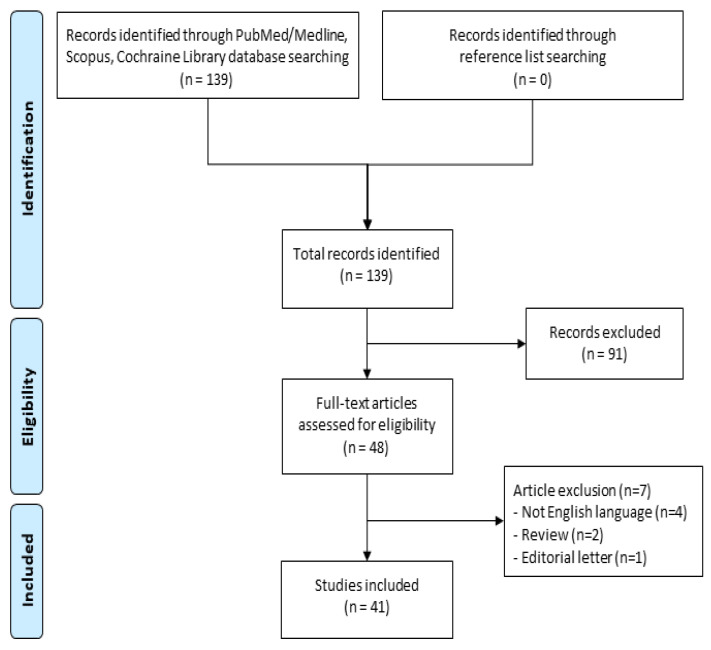
PRISMA flow diagram.

A systematic review was performed following the PRISMA (Preferred Reporting Items for Systematic Reviews and Meta-Analysis) guidelines [21], examining data from experimental studies assessing liver regeneration in pigs during the last eleven years (Figure 1). This period was chosen to avoid selection bias due to the introduction of new surgical procedures over the years.

### 2.1. Information Sources and Search

The search was conducted using PubMed, Medline, Scopus, and Cochrane Library databases up to October 2021, employing the terms: (pig OR pigs, suin OR suins, pork OR porks, swine OR swines, porcine OR porcines) AND (portal vein ligation OR PVL, portal vein embolization OR PVE, liver partition OR ALPPS OR two-stage hepatectomy OR two-stage hepatectomies OR staged hepatectomy OR staged hepatectomies) OR (hepatectomy OR hepatectomies, liver resection OR liver resections, major liver resection OR major liver resections, partial hepatectomy OR partial hepatectomies)) AND (liver regeneration OR hepatic regeneration OR regeneration OR regenerative).

### 2.2. Study Selection

All titles and abstracts of considered studies were analyzed to select those focusing on liver regeneration. After this initial process, full-text papers were screened for eligibility by two authors (L.C. and E.F.), and data were extracted using a dedicated form. The final decision on eligibility was reached by consensus between the two authors. The PubMed function ‘related articles’ was used to broaden the search, and the reference list of all eligible studies was analyzed.

### 2.3. Inclusion and Exclusion Criteria

Only studies that fulfilled the following preclinical criteria were included: (i) population: pigs, (ii) interventions: PH, PVL, PVE, ALPPS; portal vein modulation (PVM); (iii) Outcome: liver regeneration. Studies that did not fulfill inclusion criteria, conference abstracts published only as abstracts, and letters to the editor were excluded. Studies were included only when an objective evaluation of liver regeneration was presented, but the acute or survival design of the studies was not considered among the exclusion criteria.

### 2.4. Data Collection

Data were extracted through a piloted extraction form by the screening authors (L.C., E.F.). The obtained data were then compared by the two reviewers, and any inconsistencies were discussed. A third author (M.D.) was consulted, when necessary, to reach a final consensus. The following information was extracted and summarized from each study: first author and year of publication; breed, weight, and the number of pigs; type of liver resection and RLV; additional procedures; liver pre-treatments; liver functionality, regeneration, and volumetric monitoring; biochemical, histological and molecular analysis. Survival or study duration was defined as the time between the liver procedure and the death of the pig.

### 2.5. Outcomes of Interest

The primary outcome of interest was to describe the commonly used surgical models to study liver regeneration in pigs and the techniques used to estimate hepatic function and volume. The evaluation was supported and contextualized by additional data on anatomy, surgical procedures, and the hepatic regenerative process.

### 2.6. Statistical Analysis

Data were tabulated, and a descriptive analysis was performed. Categorical variables were extracted as numbers and reported as proportions.

## 3. Results

The initial search yielded 139 articles that were relevant (Figure 1). After screening titles and abstracts for irrelevance and duplication, 48 full-text articles were assessed for eligibility. Seven of these were excluded: four because they were written in a language other than English [22,23,24,25], while three were excluded for their nature as reviews [26,27] and editorial letters [28]. Finally, 41 studies were included in the qualitative analysis (Table 1) [29,30,31,32,33,34,35,36,37,38,39,40,41,42,43,44,45,46,47,48,49,50,51,52,53,54,55,56,57,58,59,60,61,62,63,64,65,66,67,68,69]. All articles were experimental studies, and they included 689 pigs. An intervention effect (or publication bias) for the analyzed outcomes was not evaluated as the sample size of each included study was too small (ranging from 5 to 36 pigs). A raw estimation of the weight was about 40 kg, with a range from 12 kg [51] to 63 kg [34].

### 3.1. Direct Hepatectomy and Staged Hepatectomy

A direct hepatectomy was performed in 32 of 36 studies that considered PH (89%). Nine articles evaluated liver regeneration as a response to PVE, PVL, and ALPPS. In particular, Asencio et al. described how a PVE performed 24 h before a 90% hepatectomy could affect the regenerative process [49], while Brige et al. used PVE to generate a 100% stenosis to be compared to a partial 20% stenosis of the portal vein [45]. Gaillard et al. proposed a new technique, making a comparison between standard permanent PVE and reversible PVE through an absorbable material, finding that repeated reversible PVE (with a second PVE treatment 14 days after the previous one) could boost liver hypertrophy more than the other “one-shot” treatments [29]. Schadde et al. [40] tried to evaluate how portal vein occlusion could improve the RLV when associated with hepatic vein ligation, finding an advantage in liver regeneration for the ladder technique and was the only one who studied the effect of vein ligation without performing a PH. Four articles considered ALPPS [44,46,47,55]. The interval time between the first and the second stage ranged from 5 days [46] to 9 days [44]. Deal et al. performed PVL, ALPPS, and “partial ALPPS” by varying degrees of parenchymal transection, demonstrating that liver hypertrophy following PVL increased following the increasing of transected parenchyma, with an inverse proportion to developing collaterals [47].

### 3.2. Study Duration

Five studies (12% of all articles) had a non-survival design [34,38,49,53,63]. The average study duration was 13 ± 11 days, ranging from 5 days [46] to 42 days (5%) [54,60], while 7 days was the most used follow-up period (32% of all studies) [30,39,40,41,47,49,50,55,62,64,65,69] (Table 1).

### 3.3. Remnant Liver Volume (RLV) and Surgical Procedures

Thirty-six studies (88% of all articles) considered hepatic resections, of which 32 were about direct hepatectomy and 4 were ALPPS [44,46,47,55]. The highest RLV was 60% of the primitive liver volume [56,61], while the lowest was 10% [38,49,52,64] (Table 2). Among the ALPPS models, only Croome et al. [55] performed an extended-left hepatectomy with a described RLV of 15–20%. The remaining three ALPPS authors [44,46,47] reported the type of resection (left hepatectomy) without quantification of RLV, and this also happened in 10 studies concerning direct hepatectomy [30,32,33,36,41,44,46,47,66,68]. Only two studies considered a hepatotoxic pre-treatment using retrorsine [42] or carbon tetrachloride (CCl4) and alcohol [56]. Regarding the PH, sixteen experiments studied liver regeneration in response to liver resection without any additional procedures, while 20 studies evaluated how the liver regenerative capacity was influenced by SCA [30,31,32,39,41,44,51,66,67,68], IRI [30,32,39,41,53,61,63,66], PVM [34,45,48,58], PVE [49], liver scaffold application [31,33], bio-artificial liver treatment [43], and application of terlipressin and octreotide [64,65]. The caudate lobe was always preserved. The most frequently applied resection was the left hepatectomy, with an associated RLV, which ranged from 20% [52] to 50% [35] when data were reported. The right lateral (RL) lobe was involved only in “small-for-size” syndrome models [38,43,49,51,52,57,59,62,64]. Twenty-three studies (64%) reported enough available data to confirm the volume of resected lobes (Table 3), showing the different types of resections with different interpretations of the amount of RLV considered.

A direct hepatectomy was performed in 32 of 36 studies that considered PH (89%). Nine articles evaluated liver regeneration as a response to PVE, PVL, and ALPPS. In particular, Asencio et al. described how a PVE performed 24 h before a 90% hepatectomy could affect the regenerative process [49], while Brige et al. used PVE to generate a 100% stenosis to be compared to a partial 20% stenosis of the portal vein [45]. Gaillard et al. proposed a new technique, making a comparison between standard permanent PVE and reversible PVE through an absorbable material, finding that repeated reversible PVE (with a second PVE treatment 14 days after the previous one) could boost liver hypertrophy more than the other “one-shot” treatments [29]. Schadde et al. [40] tried to evaluate how the portal vein occlusion could improve the RLV when associated with hepatic vein ligation, finding an advantage in liver regeneration for the ladder technique and was the only one who studied the effect of vein ligation without performing a PH. Four articles considered ALPPS [44,46,47,55]. The interval time between the first and the second stage ranged from 5 days [46] to 9 days [44]. Deal et al. performed PVL, ALPPS, and “partial ALPPS” by varying degrees of parenchymal transection, demonstrating that liver hypertrophy following PVL increased following the increasing of transected parenchyma, with an inverse proportion to developing collaterals [47].

### 3.4. Additional Procedures

#### 3.4.1. Ischemia/Reperfusion Injury

Eight articles considered IRI, as shown in Table 1. The preferred way to induce IRI was through 60 min of lasting right hepatic ischemia [30,32,39,41,61,67], while only two studies [53,63] used a 150 min Pringle maneuver. The same two authors were the only ones not to perform a PH. Shimoda et al. [61] studied how edaravone, a potent free radical scavenger, could mitigate IRI, while Arkadoupolos et al. [63] could reduce IRI through an extracorporeal plasma separation device. Only Athanasopoulos et al. [53] performed IRI to demonstrate that ischemic preconditioning could facilitate the regenerative process.

#### 3.4.2. Stem Cells Application

ADSC derived from subcutaneous porcine tissue were used six times [30,32,39,41,44,66], and the preferred site of injection was directly through the liver parenchyma. Bartas et al. [44] described the hepatic artery as the site of injection during the first stage of ALPPS, while Sang et al. [51] and Vištejnová et al. [68] preferred the portal vein to disseminate porcine mesenchymal stem cells (MSC) after the liver resection. Instead, Lim et al. [31] used hepatocyte-like cells (HLC) derived from human cord-lining epithelial cells (CLEC) applied with a collagen scaffold on the resected liver surface. Five authors [30,32,39,41,66] demonstrated that ADSC could reduce IRI (Table 1).

#### 3.4.3. Venous Blood Flow Modulation in PH

Bucur et al. [48] and Gregoire et al. [58] increased liver regeneration by applying a vascular silicon ring around the PV or the left PV, with a reduction of 20% and 45% of portal blood flow, respectively. Brige et al. [45] studied the 20% left PV flow reduction through a silk thread around the vessel. Instead, Xue et al. [69] used a silk thread to narrow the PV circumference by 1/3 and 1/2, to establish acute liver failure without liver resection. Kohler et al. performed a 30% blood flow reduction through a tourniquet around PV soon before surgery [34].

#### 3.4.4. Liver Regeneration Monitoring

As reported in Table 4, 8 authors [42,43,51,60,64,65,66,68] analyzed how cytokines and growth factors changed in response to liver resections. Nygard et al. [60] found no statistically relevant differences in IL1β, IL6, TNFα, and TGFβ after PH. Sang et al. [51] demonstrated an increment of IL1β, IL6, and TNFα when ADSCs were applied, while Inomata et al. [42] showed that retrorsine could lead to higher values of IL6 and hepatocyte growth factor (HGF). Chen et al. [43] performed a bio-artificial liver treatment using 200 g of spheroid reservoir bio-artificial liver (SRBAL) to promote liver regeneration after 85% hepatectomy, observing an increment in IL6 and TGFβ values, but not in TNFα. Instead, Brige et al. [45] demonstrated that portal vein stenosis preconditioning could determine higher values of IL6, IL10, HGF, and TNFα, even without liver resection.

##### Instrumental Functional Monitoring

Increased intracranial pressure (ICP) in patients with acute liver failure (ALF) remains a cause of morbidity and mortality after major hepatectomies. Three authors [38,43,63] considered ICP monitoring as an indirect method to state liver-correct function. Ten articles [34,36,37,40,47,48,52,57,58,59], 24% of all included studies, reported data on portal vein and hepatic artery blood flow and studied their changes after PH, or how blood flow modulation could affect liver regeneration. A dynamic liver function-hepatobiliary scintigraphy imaging was performed only by Brige et al. [45].

##### Volumetric Analysis

The most used imaging system to evaluate liver volume was the CT-scan volumetry, even if performed only by 9 authors, or 22% of all studies. Among these, three authors [38,49,51] used CT scan only to confirm a RLV < 15% of total liver volume soon after LR, not to quantify liver regeneration. One week was the most commonly used time point to evaluate the increase in liver volume after liver procedures, while two studies reported the first volumetric evaluation at postoperative day 14 [45] and 28 [29]. Bruha et al. [57] and Vištejnová et al. [68] were the only ones to perform ultrasound volumetry, while Bartas et al. [44] evaluated liver volume through MRI scan analysis on the 9th postoperative day. All other included studies did not quantify the gain in liver volume with imaging systems.

## 4. Discussion

### 4.1. Anatomical Findings in Porcine Liver

**Figure 2:**
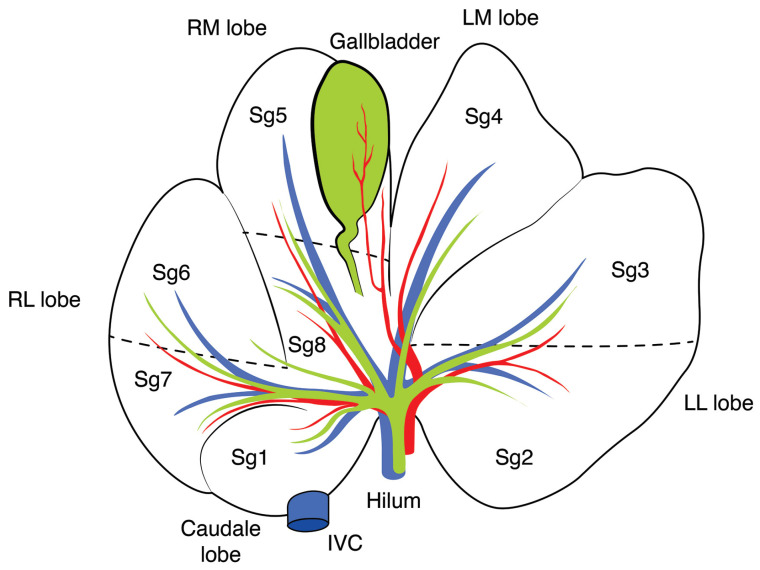
Anatomy of the porcine liver. Bile duct and gallbladder (green); artery (red); portal vein (blue); IVC = inferior vena cava. Dashed lines represent the limits between liver segments (Sg).

The porcine liver is leaf-shaped and can be divided into 5 lobes (Figure 2): right lateral (RL), right median (RM), left median (LM), left lateral (LL), and the caudate lobe (CL). It has 8 segments, similar to the human liver, each one with its arterial supply and venous and biliary drainage. As in humans, segments (Sg) were originally assigned Roman numerals, but Arabic numerals are recommended [70]. The LL lobe of the pig liver is divided into segments Sg2 and Sg3, while the RL lobe is divided into Sg6 and Sg7. The LM lobe consists of Sg4, and the RM lobe is divided into Sg5 and Sg8, while the CL corresponds to Sg1 [70]. The inferior vena cava (IVC), in Sg1, has intraparenchymal confluence with hepatic veins (HVs) [19], and this relationship makes a right hepatectomy difficult to perform. Additionally, as in humans, no branches of the bile duct, hepatic artery, or portal vein are seen to cross between the left and right hemi-liver in most cases [71]. These aspects make the pig one of the best animal models for investigating liver regeneration [72,73].

### 4.2. Surgical Procedure

According to data reported in the present review, it was possible to synthesize three main surgical procedures: (i) 50% hepatectomy removing LL and LM lobes; (ii) 70% hepatectomy (30% RLV) removing LL, LM, and RM lobes; (iii) 90% hepatectomy (10% RLV) removing LL, LM, RM lobes, and Sg6.

**Figure 3:**
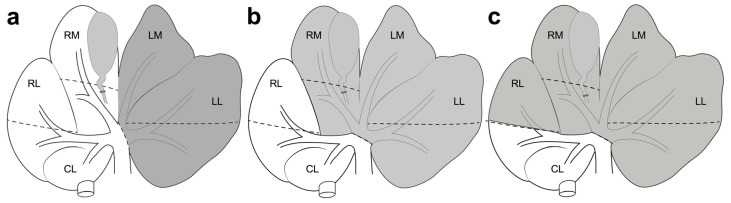
Major liver resections. The removed liver appears in grey. (**a**) 50% hepatectomy; (**b**) 70% hepatectomy; (**c**) 90% hepatectomy. Dashed lines represent the limits between liver segments (Sg).

The 50% hepatectomy (Figure 3a), considered a safe standard procedure to study the regenerative process, far from causing acute liver failure (ALF), differs from 70% hepatectomy due to the necessity to preserve RM lobe pedicle, which requires an accurate dissection of the structures of the hilum. The transection line passes between the LM and RM lobes.

From a practical point of view, the 70% hepatectomy (Figure 3b) seems to be the easiest to achieve among major hepatectomies, and the model seems to better stimulate the regenerative process, leaving the minimum amount of RLV able to avoid ALF. The transection line passes between the RM and RL lobes. Thanks to the prominent fissures of the liver, the origin of the LL, LM, and RM lobes forms a hepatic pedicle, which extends up to the vena cava and down to the liver hilum. In the open approach, the surgeon can put their fingers around this pedicle; then, the three lobes can be transected at the same time, leaving a small portion of parenchyma with the ends of intraparenchymal structures that can be sutured [74].

The 90% hepatectomy (Figure 3c) consists of a complete 70% hepatectomy, followed by the resection of Sg6 [74]. It is preferred to induce ALF, aimed at observing the role of progenitor and stem cells in the regenerative process.

The preparation of the Pringle maneuver is not mandatory but is useful to control intraoperative bleeding [75]. In humans, intermittent clamping for over 120 min (15 min of clamping combined with 5 minutes of reperfusion) is safe and effective in reducing intraoperative bleeding without impacting liver perfusion [76]. Since the blood supply of the gallbladder could come from the right or the left hepatic artery pool (from the left in 60% of the cases), [71] in the absence of preoperative diagnostics, every resection which requires the removal of the LM or RM lobe should be accompanied by cholecystectomy.

### 4.3. Recovery Time in the Regenerative Process

The time and mechanisms needed for the regenerative process change partially depending on the surgical maneuver and the RLV. For RLV > 30%, hepatic regeneration starts within minutes after the hepatectomy due to the activation of intracellular signaling pathways in hepatocytes. This phase is characterized by the hypertrophy of the hepatocytes, which is followed by a hyperplasic phase [77]. Additionally, in an RLV of about 30%, the increased portal flow creates higher shear stress on liver sinusoidal endothelial cells (LSECs), which ultimately contribute to the regenerative process, as reported in rodent models [78]. An RLV < 30% collects a portal flow that is too high for its mass compared with the arterial flow in a process called dearterialization, which dramatically reduces the hepatic artery flow [79]. This concept was the starting point for new experimental procedures for portal hemodynamic modulation. Gregoire et al., 2014 and Bucur et al., 2017 developed a portal vascular ring to improve liver regeneration by protecting liver microarchitecture [48,58]. Preziosi et al. [80] and Russel et al. [81] stated the portal flow is responsible for the delivery of molecular mediators of the regenerative process, such as WNT proteins from the LSECs, during shared stress after more than 4 h. In rodents, liver mass is restored in around seven days, while the complete restoration takes three weeks [82,83]. In humans, liver mass restores complete functionality after 3 months [84]. Sparrelid et al., 2017 observed in humans a full recovery only after 28 days post-ALPPS [85]. A detailed description of the regenerative process was reviewed in 2020 by Michalopoulos et al. [86]. The only way to assure the accuracy of the experimental procedure would be to extend the follow-up to a full recovery of the liver volume/function, and the results of this review showed that a study duration of two weeks could be adequate for many different types of hepatectomies in pigs.

### 4.4. Staged Hepatectomy and New Perspectives

PVE, introduced in humans in 1982 by Kinoshita [13], was first performed to study liver regeneration in pigs in 1999 by Duncan et al. [87]. The results of PVE in the regenerative process were consolidated in humans over the years, and a revision by Shindoh et al. reported a regeneration rate of 50% after an average of 32 days, with a post-PVE resection rate of 62–78% [88,89]. A review by Huisman et al. [26] already commented on the use of larger animals, such as pigs, in the chance to apply PVE. However, no conclusions regarding the volume increase in the future RLV were described because of the lack of standard procedures and reports [26].

The ALPPS procedure was first introduced by Schnitzbauer et al., 2012 [14], combining the PVL and the in situ splitting (ISS) of the liver. The first porcine ALPPS model was developed by Croome et al., 2015 [55]. Clinical studies stated that ALPPS allowed inducing more hypertrophy in 1 week than PVE and PVL had achieved in 3–6 weeks [90], and the LIGRO trial confirmed that the volume increase after ALPPS could allow resection within 1 to 2 weeks after the first stage [49]. There are several theories about what makes ALPPS so effective, but the exact underlying mechanisms are still the object of study. Deal et al. [47] demonstrated in pigs the relationship between more extended regeneration and the reduction of collaterals through increasing the grade of transection. This was probably due to the release of cytokines in response to the surgical trauma of parenchyma transection. Additionally, portal vein occlusion and the resulting reversal of flow in the contralateral lobe could stimulate liver regeneration [91]. However, the parenchymal connections among lobes in the porcine liver seem to be negligibly narrow and might not be enough to induce a significant inflammatory response when cut. This finding, together with the low number of porto-portal shunts in the interlobular regions of the porcine liver, let Budai et al. [27] state that pigs are less fit for ALPPS research purposes, although the procedure is indeed performable.

### 4.5. Limitations

The present systematic review has potential limitations. First, included articles presented high heterogeneity regarding population breed, size and characteristics, study duration, type of operation, and additional procedures. Second, some of the included series did not have a survival design, while others did not use imaging systems to evaluate real changes in liver volume before and after surgery. CT scans, the most used volumetric imaging system in clinical settings, were adopted in the minority of the included studies. Moreover, it seems unlikely that the same procedures with the same removed lobes and segments resulted in different volumes of RLV. Furthermore, the lack of standardization in biochemical, molecular, histopathological, and gene-expression analysis makes it difficult to extrapolate considerations that can be valid for different scenarios and countries.

The second limitation stands in the lack of experimental surgical procedures performed on the fibrotic liver, which is, instead, a common finding during hepato-biliary-pancreatic surgery. A recent retrospective clinical study by Aierken et al. showed that fibrosis could be considered a major risk factor for liver regeneration [92]. Among the included studies, only Inomata et al. and Bruha et al. tried to establish a porcine model of toxic steatohepatitis [42,56].

## 5. Conclusions

The present study provides a comprehensive overview of the existing evidence regarding surgical and interventional approaches to studying liver regeneration in pigs. The use of this animal model seems to be justified by anatomical and physiological similarities between the porcine liver and human livers. However, the lack of experimental studies reproducing the chronic pathological aspects of steatosis, fibrosis and cirrhosis still leaves lots of unsolved problems in this field. The overall revision of the available literature has unveiled an important variability in study design and endpoints. Future quantitative analyses of surgical models should be aimed at creating standards to improve scientific outputs and reproducibility. This will ultimately improve the ability of animal welfare officers to evaluate the authorizations. Therefore, we hope that preclinical research using pigs will be conducted properly and contribute to the medical sciences under the principle of animal welfare, i.e., reduction, replacement, and refinement, along with the due approval process. This systematic review could be a useful guide for all surgeons who want to study liver regeneration through large animal experimental models.

## Figures and Tables

**Table 1:** General information about selected studies.

Author	Year	Swine Breed	Weight	Study Duration (Days)	No.	Liver Resection	Additional Procedures
Hisakura et al. [62]	2010	Chinese minipig Landrace white pig	41.5 ± 9 30.1 ± 6.7	7	20	PH	-
Arkadoupolos et al. [63]	2011	-	35–40	1	12	PH	IRI
Shimoda et al. [61]	2012	-	23–26	30	12	PH	IRI
Nygard et al. [60]	2012	Norwegian landrace pig	31.7 ± 5.13	42	12	PH	-
Wang et al. [59]	2014	Bama minipig	15–20	2	20	PH	-
Gregoire et al. [58]	2014	Pietrain pig	40–50	7	24	-	PVM
Athanasopoulos et al. [53]	2015	Landrace pig	30–35	1	12	PH	IRI
Bruha et al. [56]	2015	-	-	14	20	PH	-
Nygard et al. [54]	2015	Norwegian Landrace pig	31.7 ± 5.13	42	12	PH	-
Wang et al. [57]	2015	Bama minipig	15–20	2	14	PH	-
Croome et al. [55]	2015	-	31 ± 1	7	13	ALPPS	-
Xiang et al. [52]	2016	Bama minipig	15–20	14	30	PH	-
Sang et al. [51]	2016	-	15 ± 3	14	24	PH	SCA
Bucur et al. [48]	2017	Large white pig	32.9 ± 5.3	7	17	PH	PVM
Asencio et al. [49]	2017	Minipig, Large white pig	42 ± 2	1	20	PH	PVE
Iguchi et al. [50]	2017	-	20–22	7	5	PH	-
Bartas et al. [44]	2017	Polish white pig	30–50	9	6	ALPPS	SCA
Wiederkehr et al. [46]	2017	-	-	5	10	ALPPS	-
Deal et al. [47]	2017	Yorkshire Landrace pig	-	7	12	ALPPS	-
Inomata et al. [42]	2018	Gottingen minipig	14–20	28	34	PH	-
Chen et al. [43]	2018	Large white pig	28 ± 1.2	14	18	PH	SRBAL
Ge et al. [39]	2018	Bama minipig	-	7	21	PH	IRI, SCA
Schadde et al. [40]	2018	Yorkshire landrace pig	-	7	14	-	PVM, HVL
Ge et al. [41]	2018	Bama minipig	-	7	18	PH	IRI, SCA
Brige et al. [45]	2018	Pietrain pig	-	14	14	-	PVM, PVE
Shimoda et al. [33]	2019	Large white pig	20–25	28	6	PH	Scaffolding
Zhang et al. [32]	2019	Bama minipig	25–35	7	18	PH	IRI, SCA
Fonouni et al. [36]	2019	Landrace minipig	30.2 ± 2.1	6	36	PH	-
Kohler et al. [34]	2019	Domestic minipig	56–63	1	16	PH	PVM
Bekheit et al. [37]	2019	Large white pig	32.9 ± 5.3	27	19	PH	-
Orue-Echebarria et al. [38]	2019	-	42 [39.2–49.7]	1	10	PH	-
Wittauer et al. [35]	2019	Lewe minipig	49.9 ± 2	30	7	PH	-
Jiao et al. [30]	2020	Bama minipig	20–25	21	18	PH	IRI, SCA
Lim et al. [31]	2020	Yorkshire-Dutch Landrace pig	40.5	21	16	PH	SCA, Scaffolding
Gaillard et al. [29]	2020	-	57.3 ± 5.7	28	12	-	PVE
Jo et al. [64]	2021	Large white pig	34.9 [28–39.4]	7	20	PH	Terlipressin
Jo et al. [65]	2021	Large white pig	28–40	7	18	PH	Terlipressin, Octreotide
Jiao et al. [66]	2021	Bama minipig	20–25	7	24	PH	IRI, SCA
Oldhafer et al. [67]	2021	Lewe minipig	46 ± 3	30	16	PH	HTx
Vištejnová et al. [68]	2021	Large white pig	20	14	21	PH	SCA, BDO
Xue et al. [69]	2021	Bama minipig	35–45	15	18	-	PVM

PH = partial hepatectomy; PVM = portal vein modulation; PVE = portal vein embolization; ALPPS = associating liver partition and portal vein ligation for staged hepatectomy; HVL = hepatic vein ligation; SCA = stem cell application; SRBAL = spheroid reservoir bio-artificial liver; IRI = ischemia/reperfusion injury; HTx = hepatocyte transplantation; BDO = bile duct obstruction.

**Table 2:** Remnant liver volume in partial hepatectomy.

Author	Size of Pig	Removed Lobes	RLV (%)
Shimoda et al. [33]	-	LL	-
Zhang et al. [32]	Mini	LL, LM	-
Fonouni et al. [36]	Mini	LL, LM, RM	-
Kohler et al. [34]	Mini	LL, LM, RM	30
Inomata et al. [42]	Mini	LL, LM, RL	40
Bekheit et al. [37]	Mini	LL, LM, RM	25
Bucur et al. [48]	Large	LL, LM, RM	25
Chen et al. [43]	Large	LL, LM, RM, partial RL	15
Ge et al. [39]	Large	-	-
Orue-Echebarria et al. [38]	Mini	LL, LM, RM, RL	10
Asencio et al. [49]	Mini	LL, LM, RM, RL	10
Wittauer et al. [35]	Mini, Large	LL, LM	50
Athanasopoulos et al. [53]	-	LL, LM, RM	30–20
Sang et al. [51]	Mini	LL, LM, RM, partial RL	15
Iguchi et al. [50]	Mini	LL, LM, RM	30
Bruha et al. [56]	-	LL, LM	60
Xiang et al. [52]	Mini	LL, LM, RM	20
		LL, LM, RM, 1/3RL	15
		LL, LM, RM, 2/3RL	10
Nygard et al. [54]	Large	-	40
Wang et al. [57]	Mini	LL, LM, RM, partial RL	15–10
Wang et al. [59]	Mini	LL, LM, RM, partial RL	15–10
Jiao et al. [30]	Mini	Left hepatectomy	-
Arkadopoulos et al. [63]	-	LL, LM, RM	30–25
Hisakura et al. [62]	Mini	LL, LM, RM, partial RL	20
Shimoda et al. [61]	-	LL, LM	60
Lim et al. [31]	Large	LL, LM	50
Nygard et al. [60]	Large	LL, LM, RM	40
Ge et al. [41]	Mini	Left hepatectomy	-
Bartas et al. [44]	Large	Left hepatectomy	-
Wiederkehr et al. [46]	-	LL, LM	-
Deal et al. [47]	Large	Left hepatectomy	-
Croome et al. [55]	-	LL, LM, RM, part of RL	15–20
Jo et al. [64]	Large	LLL + LML + RML + RLL	10
Jo et al. [65]	Large	LLL + LML + RML	30
Jiao et al. [66]	Mini	Left hepatectomy	-
Oldhafer et al. [67]	Mini	LLL + LML + RML	50
Vištejnová et al. [68]	-	LLL	-

LL = left lateral; LM = left medial; RM = right medial; RL = right lateral; RLV = remnant liver volume.

**Table 3:** Liver volume calculation in reported studies.

Author	Lobe Volume % (Average)							
	Left Lateral		Left Medial	Right Medial		Right Lateral		Caudate
	Sg2	Sg3	Sg4	Sg5	Sg8	Sg6	Sg7	Sg1
Xiang et al. [52]	80					13.8–16.5 (14)		4.9–7.5 (6)
Inomata et al. [42]			47.1–55.4 (51.3)	20.6–29.3 (25)		20.5–26.7 (23.4)		-
Bucur et al. [48]	75					25		
Orue-Echebarria et al. [38]	90						10	
Asencio et al. [49]	90						10	
Bruha et al. [56]	40			60				
Nygård et al. [54,60]	60					40		
Wang et al. [57,59]	75–80 (77.5)					20–25 (22.5)		
Arkadopoulos et al. [63]	70–75 (72.5)					25–30 (27.5)		
Kohler et al. [34]	70					30		
Shimoda et al. [61]	40			60				
Lim et al. [31]	50			50				
Croome et al. [55]	80–85 (82.5)						15–20 (17.5)	
Bekheit et al. [37]	75				25			
Chen et al. [43]	85						15	
Wittauer et al. [35]	50			50				
Athanasopoulos et al. [53]	70–80 (75)					30–20 (25)		
Sang et al. [51]	85						15	
Iguchi et al. [50]	70					30		
Hisakura et al. [62]	80						20	
Jo et al. [64]	90							10
Jo et al. [65]	70					30		
Oldhafer et al. [67]	50					50		

Sg = segment of the liver.

**Table 4:** Increasing in cytokine and chemokine values according to liver procedures.

Author	IL1β	IL6	IL10	HGF	TNFα	TGFβ	Liver Resection	Additional Treatments
Nygard et al. [60]	No	-	No	-	No	No	Yes	-
Sang et al. [51]	Yes	Yes	-	-	Yes	-	Yes	SCA
Inomata et al. [42]	-	Yes	-	Yes	-	-	Yes	RS
Chen et al. [43]	-	Yes	-	-	No	Yes	Yes	SRBAL
Brige et al. [45]	-	Yes	Yes	Yes	Yes	-	No	PVS
Jo et al. [64]	-	No *	-	No *	-	-	Yes	Terlipressin
Jo et al. [65]	-	No **	-	-	-	-	Yes	Terlipressin, Octreotide
Jiao et al. [66]	No	Yes	Yes	-	No	No	Yes	IRI, SCA
Vištejnová et al. [68]	-	Yes	-	-	No	Yes	Yes	SCA, BDO

* comparison between terlipressin and control group; ** comparison between terlipressin and octreotide group; SCA = stem cell application; RS = retrorsine; SRBAL = spheroid reservoir bio-artificial liver; PVS = portal vein stenosis; IL = interleukin; HGF = hepatocytic growth factor; TNF = tumor necrosis factor; TGF = transforming growth factor, BDO = bile duct obstruction.
